# Assembly of the Complex between Archaeal RNase P Proteins RPP30 and Pop5

**DOI:** 10.1155/2011/891531

**Published:** 2011-11-13

**Authors:** Brandon L. Crowe, Christopher J. Bohlen, Ross C. Wilson, Venkat Gopalan, Mark P. Foster

**Affiliations:** ^1^Department of Biochemistry, Center for RNA Biology, Ohio State University, Columbus, OH 43210-1214, USA; ^2^Ohio State Biochemistry Program, Ohio State University, Columbus, OH 43210-1214, USA

## Abstract

RNase P is a highly conserved ribonucleoprotein enzyme that represents a model complex for understanding macromolecular RNA-protein interactions. Archaeal RNase P consists of one RNA and up to five proteins (Pop5, RPP30, RPP21, RPP29, and RPP38/L7Ae). Four of these proteins function in pairs (Pop5-RPP30 and RPP21–RPP29). We have used nuclear magnetic resonance (NMR) spectroscopy and isothermal titration calorimetry (ITC) to characterize the interaction between Pop5 and RPP30 from the hyperthermophilic archaeon *Pyrococcus furiosus* (*Pfu*). NMR backbone resonance assignments of free RPP30 (25 kDa) indicate that the protein is well structured in solution, with a secondary structure matching that observed in a closely related crystal structure. Chemical shift perturbations upon the addition of Pop5 (14 kDa) reveal its binding surface on RPP30. ITC experiments confirm a net 1 : 1 stoichiometry for this tight protein-protein interaction and exhibit complex isotherms, indicative of higher-order binding. Indeed, light scattering and size exclusion chromatography data reveal the complex to exist as a 78 kDa heterotetramer with two copies each of Pop5 and RPP30. These results will inform future efforts to elucidate the functional role of the Pop5-RPP30 complex in RNase P assembly and catalysis.

## 1. Introduction

Ribonuclease P (RNase P) is a ribonucleoprotein (RNP) complex primarily responsible for cleaving the 5′ leader sequence of precursor-tRNA (pre-tRNA) molecules in all domains of life [1–3]. The RNase P RNA subunit (RPR) constitutes the Mg^2+^-dependent catalytic moiety and supports pre-tRNA cleavage on its own *in vitro *[4–6]. The bacterial RNase P holoenzyme contains one large RPR and one conserved protein (RNase P protein, RPP) that is essential for function *in vivo* [7]. The bacterial RPP aids RPR catalysis by increasing the affinity of the holoenzyme for the substrate and for the Mg^2+^ cofactor [8–11]. Eukaryal and archaeal genomes do not encode sequence homologs of the bacterial RPP [12]. Instead, eukaryal and archaeal RNase P holoenzymes comprise multiple RPPs (up to 10 in eukarya and 5 in archaea) together with an RPR [12, 13]. 

The known archaeal RPPs are homologous to eukaryal proteins Pop5, RPP21, RPP29, RPP30, and RPP38 [21–23]. Recombinantly expressed RPPs have been assembled with the *in vitro* transcribed cognate RPR to reconstitute the holoenzyme from several archaea, including *Methanothermobacter thermoautotrophicus *(*Mth*) [24, 25]* Pyrococcus horikoshii *(*Pho*) [23], *Pyrococcus furiosus *(*Pfu*) [26], *Methanocaldococcus jannaschii* (*Mja*) [25, 27], and *Methanococcus maripaludis* [22]. Reconstitution studies with *Mja*, *Mth,* and *Pfu* RNase P indicate that four RPPs work in two distinct pairs: Pop5 with RPP30 and RPP21 with RPP29 (designated Pop5-RPP30 and RPP21-RPP29, resp.). This finding is consistent with results from yeast two-hybrid assays on RPPs from other archaea (*Mth *[28], *Pho* [29]) and eukarya [*Saccharomyces cerevisiae *(*Sce*)] [30], which indicate binding interactions between these pairs of proteins. Kinetic studies continue to provide insights into the functional roles of these binary RPP complexes; for example, single-turnover experiments with *Mth* RNase P revealed that Pop5-RPP30 increases by 60-fold the rate of pre-tRNA cleavage by the RPR and that RPP21-RPP29 enhances substrate affinity by 15-fold [25]. Although various biochemical studies demonstrate functional cooperation between the RNA and protein subunits of archaeal RNase P [18, 22, 25–27, 31], high-resolution structures are critical to fully understand both the assembly of the holoenzyme and the role of RPPs in assisting RPR catalysis.

X-ray crystallography and nuclear magnetic resonance spectroscopy (NMR) have been employed to determine the structure of several archaeal RPPs, including RPP29 from *Mth* [24], *Archaeoglobus fulgidus* [32], *Pho* [33], and* Pfu* [34], Pop5 from *Pfu* [35] and *Pho* [36], *Pho* RPP30 [37], RPP21 from *Pho* [38] and *Pfu* [39] and RPP38/L7Ae [40, 41]. Structures for the RPP21-RPP29 binary complex were obtained by crystallography for *Pho* [36] and by NMR for *Pfu* [34], and of the *Pho *Pop5-RPP30 pair by crystallography [14].

**Figure 1:**
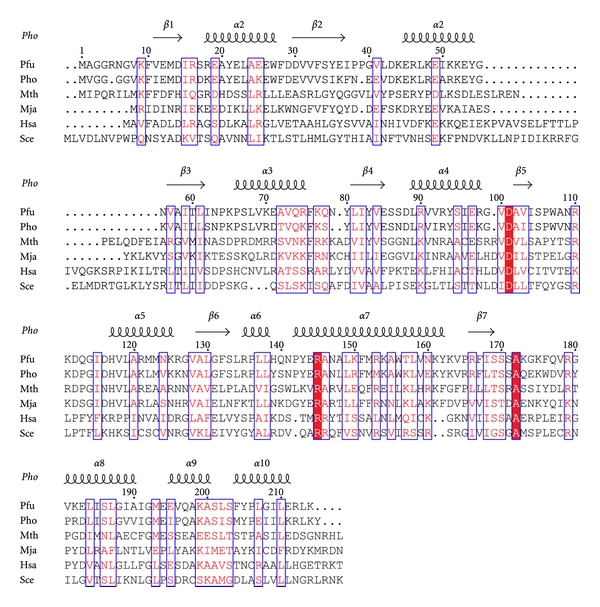
Sequence alignment of RPP30 homologs from four archaea, *Pyrococcus furiosus *(*Pfu*; NP_579643)*, Pyrococcus horikoshii *(*Pho*; NP_143706)*, Methanothermobacter thermautotrophicus *(*Mth*; NP_275831), and *Methanocaldococcus jannaschii *(*Mja*; NP_248131) and two eukaryotes *Homo sapiens *(*Hsa*; NP_006404) and *Saccharomyces cerevisiae *(*Sce*; NP_011929). Block letters indicate invariant residues and boxes indicate residues with a global similarity score >0.7. The secondary structure observed in the homologous *Pho *crystal structure [14] is depicted above the alignment. Alignment was performed using CLUSTALW [15] and colored according to similarity with a Risler scoring matrix [16] using the program ESPript [17].

We have characterized the *Pfu* Pop5-RPP30 interaction by using NMR spectroscopy and isothermal titration calorimetry (ITC). Backbone resonance assignments have been obtained for the 214-residue *Pfu* RPP30 (Figure 1) in its free state. Size exclusion chromatography (SEC) and dynamic light scattering (DLS) data indicate that the free protein is monomeric, and secondary chemical shifts are consistent with the secondary structure previously observed for *Pho *RPP30 [37]. The addition of Pop5 to uniformly ^13^C/^15^N-labeled RPP30 induces many site-specific changes in the heteronuclear NMR spectra and leads to significant broadening of most resonances. Chemical shift perturbations reveal the Pop5 binding surface on RPP30, and complement previous chemical shift perturbation maps on Pop5 [35]. NMR and ITC data indicate that these two proteins form a tight 1 : 1 complex, while SEC and DLS data indicate the *Pfu *Pop5-RPP30 complex is heterotetrameric under a range of experimental conditions. We discuss possible functional implications of these findings.

## 2. Results

### 2.1. Oligomeric State

**Figure 2:**
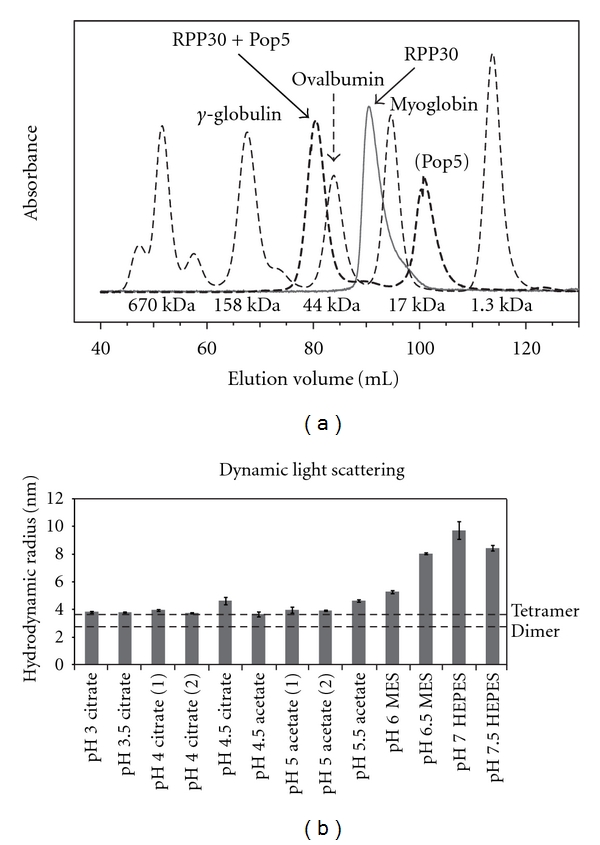
Size exclusion chromatography and dynamic light scattering of the Pop5-RPP30 complex. (a) Gel filtration chromatogram of RPP30 alone (solid), RPP30 in the presence of excess Pop5 (dashed), and molecular weight standards (dotted; thyroglobulin (670 kDa), *γ*-globulin (158 kDa), ovalbumin (44 kDa), myoglobin (17 kDa), and vitamin B_12_ (1.3 kDa)). The mixture of RPP30 with excess Pop5 yielded one peak corresponding to free Pop5, and a second peak with an apparent mass in excess of that expected for a Pop5-RPP30 heterodimer (39 kDa). (b) Measured hydrodynamic radii of the Pop5-RPP30 complex from dynamic light scattering under varying buffer conditions. The dotted lines indicate the calculated hydrodynamic radii from HYDROPRO [18] for both a heterotetramer or a heterodimer. The smallest hydrodynamic radius is consistent with formation of a heterotetramer [(Pop5-RPP30)_2_, 3.65 nm].

Size exclusion chromatography and dynamic light scattering were used to determine the oligomeric state of the Pop5-RPP30 complex (Figure 2). Free *Pfu *RPP30 eluted from the gel filtration column with an apparent molecular weight of 24 kDa, matching its monomer mass (24,495 Da). The mixture of RPP30 with an excess of Pop5 yielded two peaks, one corresponding to monomeric Pop5 (13,839 Da) and the other eluting with an apparent mass in excess of that of a heterodimer. Dynamic light scattering measurements showed that *Pfu *RPP30 and Pop5 are each monodisperse and monomeric when alone in solution (data not shown), while the 1 : 1 Pop5-RPP30 complex had a hydrodynamic radius of ~3.65 nm, corresponding to that calculated for the heterotetramer [(Pop5-RPP30)_2_] using HYDROPRO [18] (Figure 2). Although the complex seemed to aggregate at pH values above 6, it was monodisperse at lower pH values (and under a wide range of experimental conditions). These findings are in agreement with the heterotetrameric state observed in crystals of the *Pho* Pop5-RPP30 complex [14].

### 2.2. Backbone Resonance Assignments of Free RPP30

**Figure 3:**
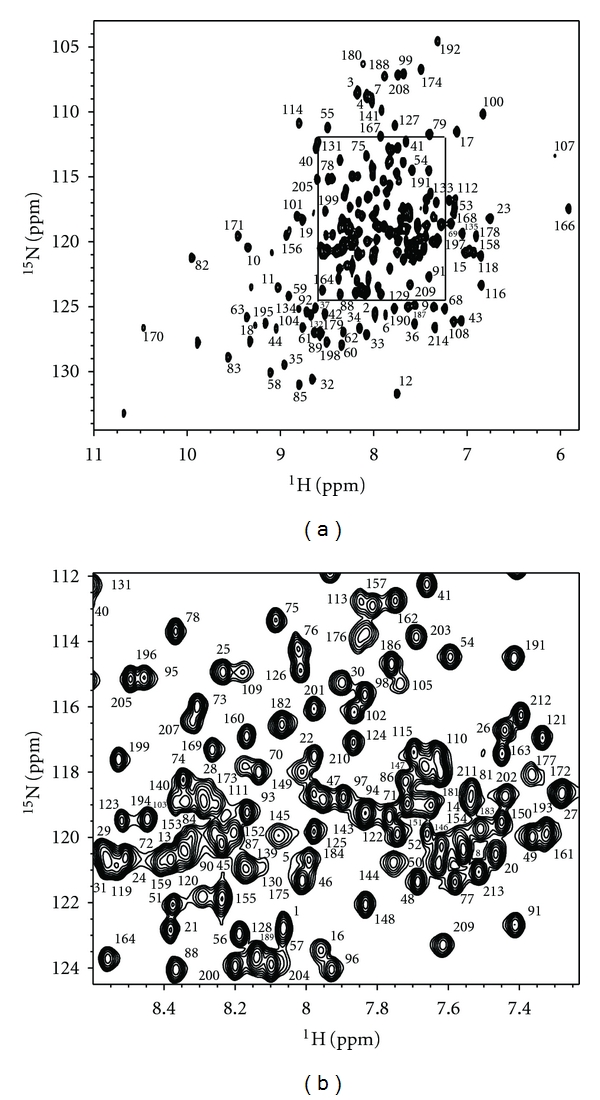
Two-dimensional ^1^H-^15^N correlation spectrum of *Pfu *RPP30. Residue numbers indicate assigned backbone amide resonances. The boxed region of the full spectrum (a) is expanded (b) for clarity.

*Pfu *RPP30 samples were highly soluble in a range of buffer conditions at 55°C, yielding well-dispersed NMR spectra indicative of a well-structured protein (Figures 3(a) and 3(b)). However, spectral quality degraded significantly at lower temperatures (e.g., 25°C), perhaps reflecting nonspecific aggregation (data not shown). Because 55°C was found to be the optimum temperature for assembly and activity of the *Pfu* RNase P holoenzyme [26] and high-quality spectra could be obtained at that temperature, all NMR experiments were performed at 55°C. 

**Figure 4:**
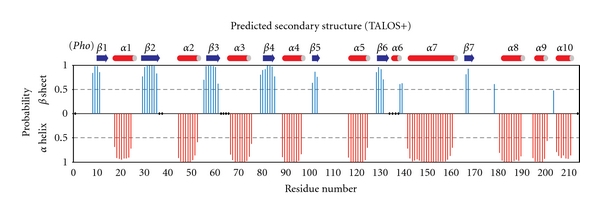
Chemical-shift-based secondary structure prediction of free RPP30 is consistent with its structural homology to *Pho* RPP30. Graph shows probability for each residue assigned to be either *β* sheet (blue above axis) or *α* helix (red below axis), as determined with the TALOS+ software [19]. Assigned secondary structure is given to each residue based on the highest probability for the residue to be either *α* Helix, *β* Sheet, or Loop. Loop residues are not indicated, while dots on the *x*-axis represent residues for which TALOS+ gave no prediction. For comparison, the secondary structure observed in crystals of the homologous *Pho *RPP30 is shown above the graph.

Of the protein's 214 native residues (Figure 1), 193 backbone amide resonances could be assigned (Figure 3). Excluding the nine proline residues, this represents approximately 94% of the assignable backbone resonances. Most unassigned residues are adjacent to prolines. Residues 137–142 comprising helix *α*6 and the *α*6-*α*7 loop are flanked by prolines 136 and 143. Intense signals were observed for the first eight N-terminal residues, suggesting that these are highly flexible in solution. Secondary structural features were predicted from analysis of backbone chemical shifts using TALOS+ [19] (Figure 4). The secondary structural features match well with the TIM barrel fold observed in the crystal structure of *Pho *RPP30, consistent with their high degree of sequence homology (68% identity, 82% similarity [15]).

### 2.3. Backbone Resonance Assignments of RPP30 in Complex with Pop5

**Figure 5:**
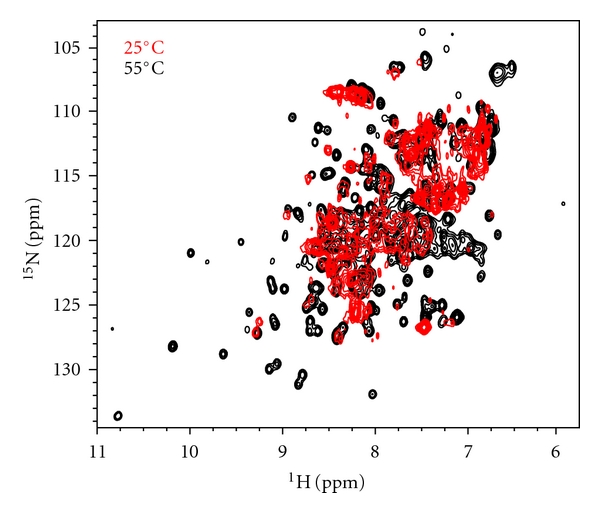
Elevated temperatures yield interpretable NMR spectra for the 78 kDa heterotetrameric *Pfu *Pop5-RPP30 complex. Overlay of two-dimensional ^1^H-^15^N correlation spectra of [U-^15^N]-RPP30 in complex with unlabeled Pop5 at 55°C (black) and 25°C (red) reveal excessive broadening at 25°C that is partly overcome at 55°C.

Compared to the free proteins, the solubility of the *Pfu *Pop5-RPP30 complex was much more sensitive to buffer conditions and mode of preparation. Data collection at lower temperatures proved intractable as spectral quality degraded significantly, while interpretable spectra could be recorded at 55°C (Figure 5). Spectra of [U-^15^N,^13^C]-RPP30 in complex with Pop5 at pH 5, 55°C remained well dispersed but highly overlapped due to increased linewidth resulting from the slower rotational correlation time of the heterotetramer. Backbone assignments of RPP30 in complex with Pop5 were obtained by tracing C^*α*^ connectivity in HNCA and HN(CO)CA spectra, using the free RPP30 assignments as a starting point, and the HNCO spectra to resolve some ambiguities. Due to increased linewidths and signal overlap in the heterotetrameric complex, only 180 backbone RPP30 amide resonances (~88%) could be assigned.

### 2.4. Pop5-Induced Chemical Shift Perturbations

**Figure 6:**
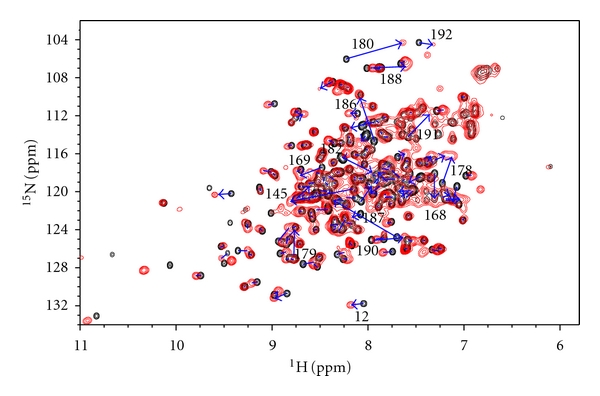
Overlay of ^1^H-^15^N correlation spectra of [U-^15^N]-RPP30 free (black) and bound to unlabeled Pop5 (red). Arrows highlight chemical shift perturbations induced by the addition of Pop5. Select perturbed amides are labeled with the residue assignment.

**Figure 7:**
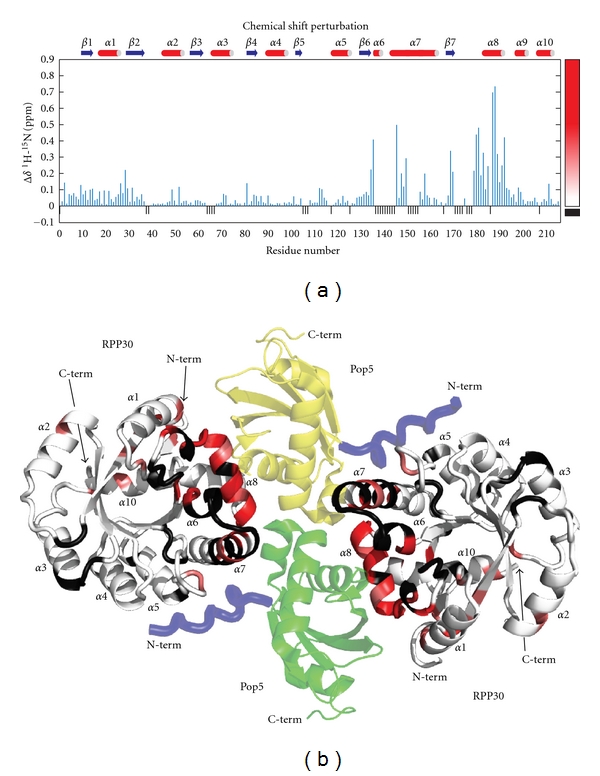
Chemical shift perturbations on RPP30 induced by Pop5 binding. (a) Secondary structure and per residue weighted average amide ^1^H and ^15^N chemical shift perturbations $\Delta \delta =\sqrt{0.5\left(\Delta \delta_{\text{H}}^{2}+{\left(\Delta \delta_{\text{N}}/5\right)}^{2}\right)}$ [20]. The color gradient used for mapping the data to the structure is shown on the right. Small black negative bars indicate residues for which the free and Pop5-bound RPP30 shifts could not be compared due to incomplete assignments or proline residues. (b) Cartoon diagram of a homology model of the *Pfu *Pop5-RPP30 complex based on the crystal structure of the complex from *Pho *[14]. Protomers of RPP30 are colored from white to red according to increasing shift perturbation. Black indicates no CSP data. The two protomers of Pop5 in the heterotetramer are green and yellow. An N-terminal segment observed in the *Pho* Pop5-RPP30 crystal structure, but not the *Pfu* Pop5 structure, is shown in blue.

The addition of unlabelled Pop5 to RPP30 induced changes to many RPP30 amide chemical shifts (Figure 6). The chemical shift perturbation pattern reflects the asymmetric interaction between each RPP30 and the two copies of Pop5 in the heterotetramer (Figure 7). In the crystal structure of the *Pho *Pop5-RPP30 heterotetrameric complex [14], the RPP30 protomers do not contact each other, but make contacts to both protomers of Pop5, burying almost 3800 Å^2^. The Pop5-RPP30 interface is dominated by *α*6, the loop between *β*7 and *α*8, as well as *α*8 of RPP30, which contact helices *α*1–*α*3 of both copies of Pop5. In the *Pho *crystal structure, additional contacts are observed between the unstructured N-terminus of Pop5 and helices *α*4 and *α*5 of RPP30 (in blue, Figure 7). While the *Pfu *NMR data are generally consistent with the *Pho *crystal structure, they indicate that only the first interface mentioned above is present in solution.

**Figure 8:**
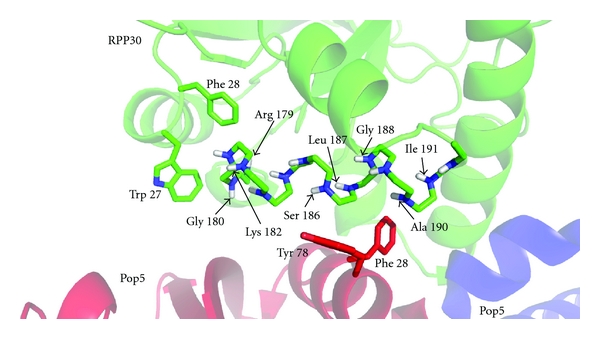
Close-up view of the Pop5-RPP30 binding interface. RPP30 is green and the two Pop5 subunits are red and blue. The backbone of *α*8 in RPP30 is shown as sticks with the amide nitrogen and proton atoms shown in blue and white, respectively. The sidechains of aromatic residues from Pop5 and RPP30 near *α*8 in RPP30 are shown as sticks. Electronic ring currents from such aromatic residues can result in large chemical shift perturbations for amides in the protein-protein interface.

Large chemical shift perturbations could be mapped to the major Pop5-RPP30 interface, comprising *α*6 and *α*8 of *Pfu *RPP30 (Figures 6 and 7). Amides of residues Gly180, Lys182, Leu187, Gly188, and Ala190 experience particularly large ^1^H shift perturbations (Δ*δ* ^1^H > 0.3 ppm; Figures 6 and 7); these large perturbations can be attributed to strong ring current effects due to packing against aromatic residues Tyr78 and Phe82 in *α*3 of Pop5, or changes in position with regard to aromatic residues Trp27 and Phe28 of RPP30 (Figure 8). Residues Arg179, Ser186, Leu187, and Ile191 each experience large perturbations of the ^15^N chemical shifts (Δ*δ* ^15^N > 2.5 ppm). These ^15^N perturbations, which are less sensitive to aromatic ring current effects [42], are likely to be caused by either changes to sidechain orientation or local backbone conformation.

In addition to this interface, the crystal structure of the *Pho* complex reveals a smaller Pop5-RPP30 interface in which the N-terminal portion of *α*7 of RPP30 contacts *α*2 of Pop5, and the N-terminal residues of Pop5 contact helices *α*4 and *α*5 of RPP30 [14]. Although the *Pfu *RPP30 backbone assignments are incomplete for the N-terminal portion of *α*7, the residues assigned in this region do show significant chemical shift perturbations, consistent with the heterotetrameric model. Unlike *α*7, all the nonproline residue of *α*4 and *α*5 are assigned but exhibit no significant chemical shift perturbations. This observation suggests that the N-terminal region of *Pfu *Pop5 does not make intimate contact with *Pfu* RPP30, which while not supportive of the heterotetramer model, is consistent with (i) the high crystallographic temperature factors observed for the N-terminus of Pop5 in the *Pho *complex [14], and (ii) the findings of complementary solution studies of labeled *Pfu *Pop5 bound to unlabeled RPP30, in which this region was observed to be largely unstructured in the absence or presence of RPP30 [35]. Thus, while the chemical shift perturbations are consistent with formation of a *Pfu *Pop5-RPP30 heterotetramer, they provide additional insight into the relative intimacy of the two sets of intermolecular contacts observed crystallographically for the *Pho* complex. The integrity of the heterotetrameric complex is further supported by hydrodynamic (see above) and thermodynamic data (see below).

### 2.5. Thermodynamics of the Pop5-RPP30 Interaction

**Figure 9:**
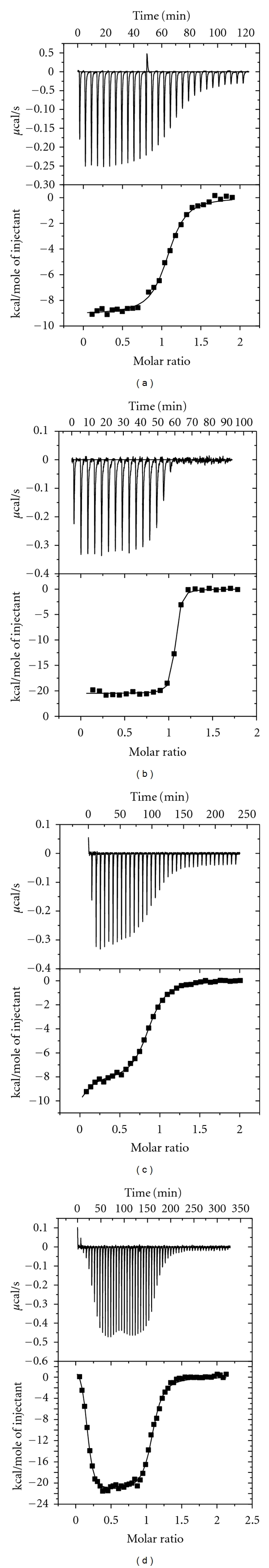
Titration calorimetry of Pop5-RPP30 binding reveals net 1 : 1 stoichiometry but higher-order binding. Top panels: Representative ITC thermograms obtained upon titration of *Pfu* Pop5 into *Pfu *RPP30 at 55°C. Bottom panels: Time-integration for each peak in the thermogram after normalizing per mol of injectant (squares). Titrations, (a, no salt) and (b, 130 mM KCl), were performed with 9.89 and 4.95 *μ*M RPP30, respectively, in the cell and can be fit by a single site binding model, with apparent dissociation constants of 124 ± 13 and 3.5 ± 0.6 nM, respectively. Experiments performed with 14.8 *μ*M RPP30 in the cell (c: no salt, d: 150 mM NaCl) yielded more complex thermograms that could only be fit by invoking two-site, or more complex binding models, consistent with higher-order assembly. Comparison of data at lower and higher ionic strength indicates binding is strongly favored by inclusion of moderate salt (e.g., 130 mM KCl) in the solutions.

The thermodynamics of the Pop5-RPP30 interaction were analyzed by isothermal titration calorimetry (ITC). The first two titrations demonstrate that the proteins form a high-affinity complex with an overall stoichiometry of 1 : 1 in the absence of other RNase P components (Figures 9(a) and 9(b)). The curves obtained under these conditions fit well to a one-site model but do not reveal a thermodynamic signature for formation of the expected 2 : 2 complex. An additional set of titrations were performed with a higher Wiseman “c” value [43] with smaller injections more carefully discern the presence of more complex binding modes. These titrations show clearly multiple binding modes with a combined stoichiometry of ~1, consistent with the expected 2 : 2 binding interaction. Although the data could be fit to a curve corresponding to a two-site binding model, given the three intermolecular interfaces observed in the complex (one Pop5-Pop5 interface, and two Pop5-RPP30 interfaces, Figure 7(b)), a more complex binding mechanism may be required to explain the thermodynamic data; exploring the details of such models is beyond the scope of the present study. Titrations performed under low ionic strength (10 mM sodium acetate) exhibited weaker binding (*K*_*d*_ of 124 nM versus 3.5 nM) and less favorable binding enthalpy than those at higher ionic strength (140 mM, 160 mM, acetate plus salt). These observations are consistent with a significant contribution to binding from the hydrophobic effect as predicted from the nature of the observed intermolecular interfaces [14, 35].

## 3. Discussion

### 3.1. Stoichiometry and Folding of RPP Binary Complexes

These biophysical investigations of the interactions between *Pfu* RNase P proteins Pop5 and RPP30 have provided definitive evidence for the formation of a tight 2 : 2 Pop5-RPP30 complex in solution in the absence of other *Pfu* RNase P components. NMR chemical shift perturbations map the binding interface of Pop5 on RPP30. Together with previously reported chemical shift perturbations of Pop5 [35], these results confirm that the crystallographic observations from *Pho* Pop5-RPP30 [14] generally hold true in solution for the closely related *Pfu* Pop5-RPP30, except that the NMR data do not reveal evidence for interaction between RPP30 and the highly conserved, relatively unstructured N-terminus of Pop5. The finding of a heterotetrameric Pop5-RPP30 pair contrasts with the observation that the other archaeal RPP pair, RPP21-RPP29, forms only a heterodimer, both in crystals and in solution [34, 36].

Another notable difference between these binary complexes is in the effect of binding on the structures of the individual proteins. NMR studies of *Pfu *RPP21 and RPP29, both individually and as a complex, revealed that disordered regions present in these proteins fold upon heterodimerization [34, 39]. These structural changes, which are essential for generating the protein-protein interface, are especially pronounced in RPP29 where 52 new (from a total of 124) backbone amide resonances, associated with residues in *α*-helices, are observed in the ^15^N-edited spectra of the RPP21–RPP29 complex. Unlike *Pfu *RPP21 and RPP29, the structures of *Pfu* Pop5 and RPP30 appear to be well formed in the absence of the other. Interestingly, despite a tight interface mediated by structured cores of the interacting partners in *Pfu* Pop5-RPP30 and RPP21–RPP29 [14, 34, 36], both complexes display one or more unstructured termini, rich in basic residues, which could potentially play a critical role in RNA binding.

### 3.2. Necessity for Formation of Binary RPP Complexes

Assembly of multicomponent complexes represents a combinatorial challenge to the cell. In this regard, preassembly of some RNase P components might confer several advantages. First, with fewer individual RNA-protein binding steps, progression towards the final, native holoenzyme is facilitated. Second, if formation of heterodimers (e.g., RPP21–RPP29) is obligatory for generating the RNA-binding platform, then the affinity of the individual subunits for the RNA could be increased significantly by employing binary complexes; this strategy may help eliminate misfolded RNAs that offer binding sites for only one protein in the dimer, or limit binding of individual proteins to unintended targets. These ideas are well illustrated by the recent studies of the RPP20–RPP25 complex, which is associated with human RNases P and MRP [44, 45]. ITC experiments revealed that the binding affinity of human RPP20–RPP25 for the P3 helix of the cognate MRP RNA increases 1000-fold relative to those observed with either RPP20 or RPP25 [44]. Consistent with this finding, a crystal structure of the *Saccharomyces cerevisiae* Pop6-Pop7 dimer (homologous to human RPP20–RPP25) bound to the RNase MRP RNA's P3 domain shows how juxtaposing POP6 and POP7 in a binary complex positions their respective RNA-binding surfaces for cooperative and specific recognition of the P3 helix [45]. 

### 3.3. Possible Roles of the Pop5-RPP30 Heterotetramer

While it is easy to appreciate the utility of pairwise interactions between RPPs, the functional significance of the *Pfu* Pop5-RPP30 heterotetramer is presently unclear. To examine this question in *Pho* RNase P, a six-residue internal deletion mutant of *Pho* Pop5 was examined for its ability to for heterotetramers and reconstitute activity [14]. The deleted residues correspond to a loop between Pop5 helices *α*1 and *α*2 that constitutes an interface between the two copies of Pop5 in the *Pho* Pop5-RPP30 heterotetramer crystal structure. Although this *Pho* Pop5 mutant assembles into a heterodimer with RPP30, it was reported to not support RPR-mediated pre-tRNA cleavage (data are not shown in [14]). However, since the mutation might affect other unintended aspects of RNase P assembly or function, it is difficult to conclude whether the heterotetrameric form of Pop5-RPP30 is indeed required for RNase P activity.

If Pop5-RPP30 were heterotetrameric in fully assembled archaeal RNase P, symmetry considerations would argue that the holoenzyme (RNA + proteins) is also dimeric. Currently, neither the stoichiometry of individual subunits nor the molecular weight of the archaeal RNase P holoenzyme is well determined. In the absence of such data, insights might be gleaned from previous findings on the eukaryal and eubacterial RNase P variants. 

Various experimental observations hint towards higher-order structure in eukaryotic RNase P. Yeast two-hybrid studies have established self-association of different human/yeast RPPs, leading to expectations that multiple copies of some of these subunits might be present in their respective native holoenzymes [30, 46]. Protein quantitation by SYPRO Ruby fluorescence staining in RNase MRP, an RNP complex involved in rRNA and mRNA processing and shares several protein subunits with RNase P [47], suggests that multiple copies of nine of the ten protein subunits are associated with the MRP RNA [48]. Additional experimental evidence is needed to corroborate and extend these stoichiometric estimates.

Hints at functional dimerization have also emerged from biochemical and biophysical studies of eubacterial RNase P. Small angle X-ray scattering (SAXS) studies revealed that although the *Bacillus subtilis* RNase P RNA exists as a monomer in solution, addition of its cognate protein cofactor results in formation of a holoenzyme dimer [49, 50]. The oligomerization state is sensitive to the ionic conditions, with dimers and monomers favored at low and high ionic strength, respectively. Moreover, at low ionic strength, the holoenzyme dimer was observed to dissociate upon addition of a monomeric pre-tRNA substrate, while a mixture of monomeric and dimeric ES complexes were observed upon addition of a dimeric substrate. 

While there is no direct experimental evidence for presence of the RNase P dimer *in vivo*, the *K*_*D*_ value of 50 nM (at 0.1 M NH_4_Cl) reported for the dimerization of *B. subtilis* RNase P would permit formation of the dimer at the estimated intracellular concentrations of bacterial RNase P (up to 42 nM in *B. subtilis *[50] and 680 nM in *E. coli *[51]). Among possible payoffs from a holoenzyme dimer, Pan and coworkers considered both cooperative substrate binding and increased processivity during maturation of polycistronic transcripts containing multiple tRNAs (e.g., the largest tRNA operon in *B. subtilis* which codes for 21 tRNAs). Whether polycistronic substrates accumulate at steady state is unclear, given the concerted actions of various nucleases (e.g., RNase E in some bacteria [52, 53]) that simplify the task of RNase P by generating monomeric intermediates from polycistronic precursors. 

Oligomerization of RNase P might confer gains in addition to processivity. The half-life of the RNA moiety might be enhanced in a higher-order RNP complex. Or, certain oligomeric states, even if comprised of only the RPR and a partial suite of RPPs, might represent either inactive forms that prevent nonspecific cleavage of unintended cellular RNAs or intermediates primed to assemble into the holoenzyme. Further studies are clearly needed to gain a full understanding of the functional relevance of oligomeric forms of RNase P.

## 4. Materials and Methods

### 4.1. Protein Preparation

Unless otherwise noted, Pop5 and RPP30 refer to the *Pfu* proteins throughout. Unlabelled Pop5 (NP_579107) and RPP30 (NP_579643) samples were prepared as described previously [35]; the Cys72Ser variant of Pop5 was used in this study due to its decreased tendency to aggregate. Uniformly doubly labeled RPP30 ([U-^13^C, ^15^N]-RPP30) was purified from cells grown in M9 minimal medium supplemented with 1% (v/v) Eagle Basal Vitamin Mix (Life Technologies, Gaithersburg, MD) [54], containing 1 g/L ^15^N ammonium chloride as the sole nitrogen source and 3 g/L ^13^C glucose as the sole carbon source. The final RPP30 protein used includes three additional residues (Gly-Glu-Phe) at the N-terminus, which remain after TEV protease cleavage of a (His)_6_-maltose binding protein tag used for affinity purification. Labeled RPP30 was dialyzed into NMR buffer [10 mM sodium acetate, pH 5, 0.02% (w/v) sodium azide] and concentrated to 1 mM. D_2_O was added to 5% (v/v). Unlabeled proteins were dialyzed at room temperature for 3 days into NMR buffer to ensure complete equilibration in preparation for ITC.

### 4.2. Gel Filtration

Size exclusion chromatography was performed with each protein alone and with RPP30 mixed with an excess of Pop5. The proteins were dialyzed into NMR buffer with 500 mM NaCl and run on a Hiload 16/60 Superdex-200 gel filtration column (GE healthcare).

### 4.3. Dynamic Light Scattering

Dynamic light scattering (DLS) was used as a preliminary screen to identify conditions that produced favorable NMR spectra. Samples for DLS were prepared by dialyzing 100 *μ*L of each protein (90 *μ*M) into each buffer tested (Figure 2). Stoichiometric amounts of Pop5 were then slowly pipetted into RPP30 resulting in a 45 *μ*M sample of the complex. Each sample was filtered through a 0.22 *μ*m filter (Costar Spin-X Centrifuge Tube Filter, Corning Inc.) and analyzed with a DynaPro-801 Molecular Sizing Instrument (Protein Solutions, Inc.). The behavior of each sample was examined in terms of its estimated hydrodynamic radius, as well as a low polydispersity over radius (polyD/r). Polydispersity is representative of the particle size distribution width, thus a low polyD/r value indicates a homogeneous mixture. The buffer conditions sampled ranged from pH 3 to 8 in increments of 0.5; reported values are the mean and standard deviation from 18 measurements (Figure 2). DLS measurements in salt concentrations from 0 to 1 M NaCl showed that the proteins had a tendency to aggregate above 250 mM NaCl (data not shown). Below 250 mM NaCl, DLS measurements over a range of protein concentrations from 50 to 500 *μ*M were consistent with a single, heterotetrameric species.

### 4.4. Preparation of the Pop5-RPP30 Complex

The NMR sample of labeled RPP30 in complex with unlabeled Pop5 was prepared by dialyzing each protein separately into a buffer containing 10 mM sodium acetate (pH 5) with 0.02% (w/v) sodium azide. RPP30 and Pop5 were concentrated to 575 and 1,575 *μ*M, respectively. Equimolar amounts of each protein were mixed slowly by use of Spectra/Por CE dialysis tubing 100,000 MWCO (Spectrum Laboratories, Inc). The resulting solutions yielded a Pop5-RPP30 complex at a concentration of 425 *μ*M in buffer containing 10 mM sodium acetate (pH 4), 0.02% (w/v) sodium azide. For NMR experiments, D_2_O was added to 5% (v/v). Free U-[^13^C,^15^N]-RPP30 (0.5 mM) was prepared in the same buffer for direct comparison of NMR spectra of RPP30, free and bound to Pop5.

### 4.5. NMR Spectroscopy

Triple resonance TROSY [55] spectra (HNCO, HNCA, HNCACB, and CBCA(CO)NH) [56] were recorded on U-[^13^C,^15^N]-RPP30 at pH 5 for backbone resonance assignments. Triple resonance spectra without the TROSY effect (HNCO, HNCA, HN(CO)CA, CBCA(CO)NH) [56] were recorded on U-[^13^C, ^15^N]-RPP30 in complex with unlabeled Pop5 at pH 4 for backbone assignments of the complex. All NMR experiments were performed at 55°C on a Bruker DRX-600 spectrometer equipped with a cryogenically cooled triple resonance single-axis gradient probe. Data were processed with NMRPipe [57] and analyzed using NMRViewJ [58]. Backbone assignments of free RPP30 were obtained by manual inspection of the data, with the assistance of the Probabilistic Interaction Network of Evidence (PINE) algorithm [59]. Pop5-induced chemical shift perturbations on RPP30 were quantified using the chemical shift differences of amide ^1^H and ^15^N resonance frequencies as $\Delta \delta =\sqrt{0.5\left(\Delta \delta_{\text{H}}^{2}+{\left(\Delta \delta_{\text{N}}/5\right)}^{2}\right)}$ [20]. Assignments for free and Pop5-bound RPP30 have been deposited in the BioMagResBank (BMRB: http://www.bmrb.wisc.edu/) with accession numbers 17189 and 17190, respectively.

**Isothermal Titration Calorimetry, (ITC)**

Protein concentrations were determined by absorbance at 280 nm in 20 mM sodium phosphate (pH 6.5), 6 M guanidium hydrochloride using molar extinction coefficients predicted from their amino acid sequences [60]: RPP30 *ε*_*280*_ = 28,590 M^−1^ cm^−1^, Pop5 *ε*_*280*_ = 19,750 M^−1^ cm^−1^.

The first set of titrations performed was Pop5 (87.2 *μ*M) titrated into RPP30 (9.89 *μ*M and 4.95 *μ*M) (Figures 9(a) and 9(b), resp.). All titrations were performed at 55°C on a MicroCal VP-ITC microcalorimeter, with the first injection being 5 *μ*L and all subsequent injections being 10 *μ*L with 240 seconds between injections; the first 5 *μ*L injection was discarded from analysis. The heat of dilution was estimated by averaging the last 3 to 5 nonreacting injections in which excess Pop5 was injected into saturated RPP30. The titrations were carried out in the absence (Figure 9(a)) and presence of 130 mM KCl (Figure 9(b)). Data were fit to a one-site model with the Origin software v. 7 (MicroCal, Inc.) to obtain the binding stoichiometry *n*, association constant *K*_*A*_, and binding enthalpy ΔH. Although these data revealed a net 1 : 1 stoichiometry, they could not distinguish a 1 : 1 heterodimer from the 2 : 2 stoichiometry of the heterotetramer.

To reveal the thermodynamic signature of forming a heterotetrameric complex, a second set of titrations was performed while aiming to vary the Wiseman “c” value [43]. When Pop5 (148 *μ*M) was titrated into RPP30 (14.8 *μ*M) in the absence of salt (Figure 9(c)), the first injection was 4 *μ*L and all subsequent injections were 7.5 *μ*L, with 400 s between injections. The titration with 150 mM sodium chloride (Figure 9(d)) had a first injection of 2.5 *μ*L and all subsequent injections of 5 *μ*L with 350 s between injections. The injection volume was decreased in order to increase the likelihood of observing multiple binding sites. Data were fit to a two-site model by Origin software v. 7 (MicroCal, Inc.).

### 4.6. Homology Modeling

A homology model of *Pfu *Rpp30 was obtained by threading its amino acid sequence into the coordinates of *Pho* RPP30 (PDB entry 2CZV, chain b) [14] using SWISS-MODEL [61]. A model of the *Pfu *heterotetramer was assembled by aligning the coordinates of *Pfu *Pop5 [35] with the corresponding coordinates of *Pho *Pop5 in the same coordinate set (2CZV chains c, d). Superposition and surface calculations were performed with Pymol (http://www.pymol.org/).
